# Hyperactivation of Posterior Default Mode Network During Self-Referential Processing in Children at Familial High-Risk for Psychosis

**DOI:** 10.3389/fpsyt.2021.613142

**Published:** 2021-02-09

**Authors:** Guusje Collin, Clemens C. C. Bauer, Sheeba Arnold Anteraper, John D. E. Gabrieli, Elena Molokotos, Raquelle Mesholam-Gately, Heidi W. Thermenos, Larry J. Seidman, Matcheri S. Keshavan, Martha E. Shenton, Susan Whitfield-Gabrieli

**Affiliations:** ^1^Department of Brain and Cognitive Sciences, McGovern Institute for Brain Research, Massachusetts Institute of Technology, Cambridge, MA, United States; ^2^Department of Psychiatry, Beth Israel Deaconess Medical Center, Harvard Medical School, Boston, MA, United States; ^3^Department of Psychiatry, University Medical Center Utrecht Brain Center, Utrecht, Netherlands; ^4^Department of Psychology, Northeastern University, Boston, MA, United States; ^5^Department of Psychology, Suffolk University, Boston, MA, United States; ^6^Psychiatry Neuroimaging Laboratory, Department of Psychiatry, Brigham and Women's Hospital, Harvard Medical School, Boston, MA, United States

**Keywords:** schizophrenia, self-referential processing, default mode network, familial high-risk, psychosis

## Abstract

Patients with schizophrenia spectrum disorders show disturbances in self-referential processing and associated neural circuits including the default mode network (DMN). These disturbances may precede the onset of psychosis and may underlie early social and emotional problems. In this study, we examined self-referential processing in a group of children (7–12 years) at familial high risk (FHR) for psychosis (*N* = 17), compared to an age and sex-matched group of healthy control (HC) children (*N* = 20). The participants were presented with a list of adjectives and asked to indicate whether or not the adjectives described them (self-reference condition) and whether the adjectives described a good or bad trait (semantic condition). Three participants were excluded due to chance-level performance on the semantic task, leaving *N* = 15 FHR and *N* = 19 HC for final analysis. Functional MRI (fMRI) was used to measure brain activation during self-referential vs. semantic processing. Internalizing and externalizing problems were assessed with the Child Behavior Checklist (CBCL). Evaluating main effects of task (self > semantic) showed activation of medial prefrontal cortex in HC and precuneus/posterior cingulate cortex (PCC) in FHR. Group-comparison yielded significant results for the FHR > HC contrast, showing two clusters of hyperactivation in precuneus/ PCC (*p* = 0.004) and anterior cerebellum / temporo-occipital cortex (*p* = 0.009). Greater precuneus/PCC activation was found to correlate with greater CBCL internalizing (*r* = 0.60, *p* = 0.032) and total (*r* = 0.69, *p* = 0.009) problems. In all, this study shows hyperactivity of posterior DMN during self-referential processing in pre-adolescent FHR children. This finding posits DMN-related disturbances in self-processing as a developmental brain abnormality associated with familial risk factors that predates not just psychosis, but also the prodromal stage. Moreover, our results suggest that early disturbances in self-referential processing may be related to internalizing problems in at-risk children.

## Introduction

Schizophrenia spectrum disorders are widely considered to be disorders of brain development, even though their most characteristics symptoms do not appear until the first manifestation of psychosis, typically in adolescence or early adulthood (1, 2). The neurodevelopmental model of schizophrenia accounts for observations that children who have an increased risk to develop psychosis show delays in neuromotor, language, and cognitive development and socio-emotional problems (3, 4). These findings suggest that psychosis may be the end result of abnormal neurodevelopmental processes that start years before illness onset (5).

There are various theories as to why psychotic symptoms take so long to manifest when the underlying etiology is likely neurodevelopmental in nature. On such theory is the “two-hit” hypothesis, which posits that early genetic and environmental risk factors may disrupt certain aspects of brain development, but only to the extent that they render an individual vulnerable to a “second hit.” This second hit, such as exposure to trauma or substance abuse, would then be the catalyst for the actual manifestation of the illness (6). Another theory is that early, subtle, deficits in brain development may progress beyond a threshold critical for the expression of psychotic symptoms as a function of otherwise normal neuro-maturational events (7). For example, early dendritic spine deficits may be aggravated by (in itself normal) synaptic pruning in adolescence and only then become detrimental to brain and cognitive functioning.

To clarify the developmental trajectory leading up to the manifestation of psychosis, we need to understand the timing of brain and socio-cognitive abnormalities that have been observed in patients with established illness. One consistent finding in schizophrenia is that patients show disturbances in self-related processing, which have been attributed to abnormal processing of the default-mode network (DMN) and related structures (8–11). The DMN comprises medial prefrontal cortex (MPFC), precuneus/posterior cingulate cortex (PCC), and lateral parietal cortex and the cortical midline sections in particular—i.e., MFPC and precuneus/PCC—are thought to be involved in internally focused processes (12, 13). Disturbances in self-referential processing have been linked to delusions, hallucinations, poor insight, and social deficits (14) and have been hypothesized to “represent an early, premorbid (i.e., pre-prodromal) indicator of schizophrenia risk that results from abnormalities of the structure and function of the neural circuitry of self” (14). However, there are few neuroimaging studies in pre-adolescent FHR children, and none that have directly studied neuroimaging correlates of self-referential processing.

To address this knowledge gap, we investigated a group of children between 7 and 12 years of age who are at Familial High Risk (FHR) for psychosis because they have a parent or sibling affected by psychotic illness. A data-driven analysis of resting-state functional MRI (fMRI) in this sample revealed abnormal functional connectivity of posterior superior temporal gyrus with a set of language and DMN-associated brain regions (15). In the current study, we studied brain activation during self-referential processing using a commonly used fMRI self-reference paradigm that was adapted to meet the developmental level of children in this age range. Based on theoretical accounts of self-referential processing as a premorbid indicator of schizophrenia (referenced above) and previous findings of DMN abnormalities in unaffected relatives of patients with schizophrenia (16, 17), we hypothesized to find hyperactivity of DMN midline regions during self-referential processing in FHR children relative to HC. If the current study confirms this hypothesis, it would confer important new information about the timing of DMN changes in psychotic illness development.

Moreover, as FHR children show elevated levels of behavioral problems (18, 19), we set out to assess putative disturbances in self-related processing for associations with behavioral data. Childhood behavioral problems are generally classified into two broad categories: internalizing and externalizing problems (20). Disturbances in self-referential processing may relate specifically to internalizing problems. For example, excessive worrying or rumination has been related to negative self-referential processing and a heightened focus on negative emotional states, in association with activation of core DMN regions (21, 22). Moreover, specific behavioral problems, including withdrawn behavior and thought problems, have been associated with increased risk for developing psychosis (23, 24). This association may also be mediated by self-processing deficits, as difficulties in distinguishing self-relevant from self-irrelevant information may render the perception of neutral environmental stimuli as abnormally salient and thereby contribute to the development of psychosis (-like) symptoms (25). Therefore, we aimed to investigate how putative disturbances in self-related processing relate to childhood behavioral indicators of future psychopathology.

## Materials and Methods

### Participants

A total of 37 children, ages 7–12 years, participated in this study. Our sample included 17 FHR children and 20 age/sex-matched healthy control (HC) children. Three children (two FHR, one HC) showed chance-level performance on the behavioral task (see behavioral data analysis for details) and were therefore excluded from further analysis. The remaining 15 FHR had at least one first-degree relative affected by psychotic illness (see Table 1 for details) and originated from a total of 10 families, including three sibling pairs and one set of three siblings. The remaining 19 HC originated from 16 families and included three sibling pairs. Exclusion criteria for HC included a family history of psychosis or bipolar disorder in first-degree relatives, personal lifetime history of a major mental disorder, and a lifetime history of antipsychotic treatment. Exclusion criteria for FHR participants included current or recent use (within the last 30 days) of anti-psychotic medication. Current or recent use (defined as within four half-lives of the concerned medication) of any other psychotropic medication was an exclusion criterion for all participants. Intellectual disability (i.e., IQ <70) was also an exclusion criterion for both groups. Participants were recruited at the Department of Psychiatry of Beth Israel Deaconess Medical Center (BIDMC) in Boston. BIDMC's Institutional Review Board (IRB) approved the study. Parental consent to participate in the study was obtained for all participants, and the participants themselves provided assent.

**Table 1:** Demographic and clinical information.

	**FHR** **(*N* = 15)**	**HC** **(*N* = 19)**	**Statistics**
Age in years, mean (sd) [range]	9.6 (2.0) [7.0 – 12.4]	9.3 (1.7) [7.2 – 12.2]	*F*_(1, 33)_ = 0.3, *p* = 0.572
Sex, M/F	5 / 10	9 / 10	*χ^2^* = 0.7, *p* = 0.409
WISC IQ, mean (sd) [range]	102.9 (15.0) [73 – 132]	111.5 (17.0) [81 – 153]	*F*_(1, 33)_ = 2.5, *p* = 0.126
**DSM-diagnosis**			*χ^2^* = 9.4, *p* = 0.025
ADHD	4	0	
ADHD/ODD	2	0	
No diagnosis	6	14	
Data missing	3	5	
**CBCL scores**			
Internalizing problems, mean (sd) [range]^a, b^	6.9 (8.6) [0 – 33]	1.8 (2.5) [0 – 10]	*F*_(1, 32)_ = 6.0, *p* = 0.020
Externalizing problems, mean (sd) [range]^a, b^	8.1 (8.6) [0 – 34]	1.7 (4.1) [0 – 18]	*F*_(1, 32)_ = 6.9, *p* = 0.013
Total problems, mean (sd) [range]^a, b^	31.7 (29.4) [2 – 119]	8.8 (12.1) [0 – 54]	*F*_(1, 32)_ = 9.5, *p* = 0.004
Affected relative, parent / sibling	10 / 5	N/A	
Diagnosis proband, SCZ / SA / other	8 / 5 / 2	N/A	
Motion, scrubbed volumes (%) across 2 runs, mean (sd) [range]	7.2 (3.6) [1.7 – 15.5]	7.1 (3.8) [1.1 – 17.1]	*F*_(1, 33)_ = 0.0, *p* = 0.993

Statistical comparisons were performed using analysis of variance (ANOVA) for continuous and chi-squared tests for categorical variables.

^a^Data missing for one participant.

^b^There was one major outlier in Childhood Behavior Checklist (CBCL) scores (i.e., all > 3 sd above the mean) (Supplementary Figure 1). Group-effects were highly similar if this participant was excluded (all p ≤ 0.02).

*ADHD, attention deficit hyperactivity disorder; ODD, oppositional defiant disorder*.

### Clinical Evaluation

#### Clinical Assessment

All HC and FHR participants were assessed for current psychiatric disorders using the SCID for Childhood Diagnoses (Kid-SCID) (26). Clinical diagnoses of affected family members of FHR participants were confirmed using the Structured Clinical Interview for DSM-IV (SCID) (27), combined with an evaluation of their medical history and interviewing at least one informant, and determined in consensus during meetings attended by senior clinicians (LJS, MSK, RMG).

#### Cognitive Assessment

Overall IQ was estimated using subtests from all four domains of the Wechsler Intelligence Scale for Children-IV (WISC-IV) (28).

#### Behavioral Assessment

Behavioral problems were assessed using the Child Behavior Checklist (CBCL) (29). The CBCL includes eight subscales (anxious/depressed, withdrawn, somatic complaints, social problems, thought problems, attention problems, rule breaking, and aggressive behaviors) and two broadband domains: internalizing problems (consisting of withdrawn, somatic complaints, and anxious/depressed subscales) and externalizing problems (consisting of rule breaking and aggressive behavior scales).

Demographic, clinical, cognitive, and behavioral variables are summarized in Table 1.

### fMRI Self-Reference Task

To assess self-referential processing, participants performed a self-reference task during fMRI, in which they were presented with two lists of 24 adjectives, describing positive (e.g., friendly, honest) and negative (e.g., boring, rude) traits. The lists were presented in six blocks of 4 words each, alternating between a semantic and a self-reference condition. The average number of letters and syllables per word was similar between valences and across the lists.

#### Semantic Condition

In the semantic condition, participants were asked to determine for each presented word, whether it described a good or bad trait, using a two-alternative forced-choice button press (“good” or “bad”).

#### Self-Reference Condition

In the self-reference condition, participants were asked to determine for each presented word whether it described them or not, using a two-alternative forced-choice button press (“me” or “not me”).

### Behavioral Data Analysis

#### Semantic Condition

Semantic performance, measured as the percentage of correct responses during the semantic condition, was assessed for each participant to gauge task engagement and performance. Three participants (i.e., one HC and two FHR, mean age of 8.1 years) were found to perform around chance-level, responding correctly to an average (sd) [range] of 58.3% (4.2%) [54.2–62.5%] of semantic stimuli, and were excluded from further analysis.

#### Self-Reference Condition

For each participant, the percentage of semantically good and bad adjectives associated with “me” were computed as a measure of positive and negative self-appraisal, respectively.

#### Statistical Analysis

Semantic performance and positive and negative self-appraisal were compared between groups using Analysis of Variance (ANOVA) and assessed for correlations with clinical variables (i.e., CBCL scores and IQ) in each group separately.

### Imaging

#### Image Acquisition

MRI scans were acquired on a Siemens Magnetom Trio 3T scanner, using a commercially available 32-channel radio frequency brain array coil (Siemens AG, Healthcare Sector, Erlangen, Germany). A single-shot gradient-echo sequence was used to collect task functional MRI (task-fMRI) data (88 functional volumes per run, TR = 2,000 ms, TE = 30 ms, 3 mm isotropic voxels, duration 2'56” per run). A 3-dimensional high-resolution T1-weighted structural scan was collected for anatomical localization (MPRAGE, TR = 2,530 ms, TE = 3.39 ms, inversion time = 1,100 ms, FA = 7°, voxel size 1.3 × 1 × 1.3 mm^3^).

#### Image Preprocessing

Preprocessing of imaging data was performed with FSL Version 5.0.9 (FMRIB, Oxford, UK) (30) and included brain extraction using FSL's BET (Brain Extraction Tool) (31), normalization to MNI space, and motion correction using MCFLIRT (intra-modal motion correction tool) (32). The remaining fMRI signals were spatially blurred with a 5 mm full-width-at-half-maximum (FWHM) Gaussian kernel. A subject-dependent number of individual nuisance regressors for removing outlier time points were created with FSL's Motion Outliers tool, which uses the spatial root mean square of the data after temporal differencing (DVARS) to identify intensity outliers, using the standard boxplot outlier threshold set in FSL. The threshold for exclusion was set at >20% of scans identified as outliers; none of the participants exceeded this threshold.

#### fMRI Analysis and Experimental Design

Analysis of fMRI task-data was performed using FSL FEAT (FMRI Expert Analysis Tool) Version 6.00 (33). Brain activation for self-reference vs. semantic processing (“self-ref > semantic”) was calculated separately for each run of each participant. To this end, first-level GLM analyses included two regressors modeling the self-reference and semantic condition per run. These regressors were modeled as a boxcar function with values one, respectively, convolved with a single gamma hemodynamic response function. The global mean time series of each preprocessed run, six motion parameters, and the motion outliers were added to the GLM as nuisance regressors. The resulting individual contrast images were entered into a second level fixed-effects analysis including both runs for each participant. In third-level analysis, mixed effects Analysis of Variance tests were conducted to assess main effects of task and group (FHR > HC and HC > FHR) were conducted. Significant effects were followed up with *post-hoc* testing. All analyses were performed on a whole-brain level. Results are reported with *Z*-statistic (Gaussianised T/F) images thresholded using clusters determined by *Z* > 3 and a (corrected) cluster significance threshold of *p* = 0.01 (34, 35) after correcting for age and sex.

### Associations With Clinical Measures

Group-differences in brain activation during self-referential processing were assessed for correlations with clinical variables. To this end, signal intensity values from each participant's contrast parameter estimates for the significant clusters were extracted using Featquery (36) and assessed for correlations with CBCL scores and IQ. In addition, exploratory analyses were performed to assess correlations with CBCL subscale scores and associations with emotion regulation strategies measured with the Emotion Regulation Questionnaire (ERQ) (37, 38) were assessed in a *post-hoc* analysis.

## Results

### Group Characteristics

Group-comparisons on demographic characteristics confirmed that the groups were well-matched for age and sex. Average IQ was almost nine points lower in FHR than HC, but this group-difference was not statistically significant (*p* = 0.13), possibly due to sample size. Approximately 40% of FHR had a DSM-diagnosis (mainly ADHD), while there were no DSM-diagnoses in the HC group (χ^2^ = 10.2, *p* = 0.02). FHR showed more internalizing, externalizing, and total problems on CBCL (all *p* ≤ 0.02). See Table 1 and Supplementary Figure 1 for details.

### Behavioral Results

There were no significant group-differences in semantic performance or positive and negative self-appraisal (Table 2). In FHR, semantic performance and negative self-appraisal were associated with IQ, such that higher IQ was associated with better semantic performance (*r* = 0.63, *p* = 0.013) and lower negative self-appraisal (*r* = −0.59, *p* = 0.018) (Supplementary Figure 2).

**Table 2:** Behavioral results.

	**FHR** **(*N* = 15)**	**HC** **(*N* = 19)**	**Statistics**
**Semantic condition**			
Semantic performance (%)	89.1 (9.9) [66.7 – 100]	89.7 (7.8) [70.8 – 100]	*F*_(1, 33)_ = 0.0, *p* = 0.864
**Self-reference condition**			
Positive self-appraisal (%)	87.6 (12.1) [66.7 – 100]	89.7 (9.8) [66.7 – 100]	*F*_(1, 33)_ = 0.3, *p* =0.565
Negative self-appraisal (%)	20.0 (15.5) [0 – 58.3]	18.7 (19.0) [0 – 66.7]	*F*_(1, 33)_ = 0.0, *p* = 0.829

*Semantic performance, measured as the percentage of correct responses during the semantic condition, and positive- and negative self-appraisal, measured as the percentage of semantically good and bad adjectives associated with “me,” per subject group*.

### fMRI Results

#### Main Effect of Task per Group

**Figure 1:**
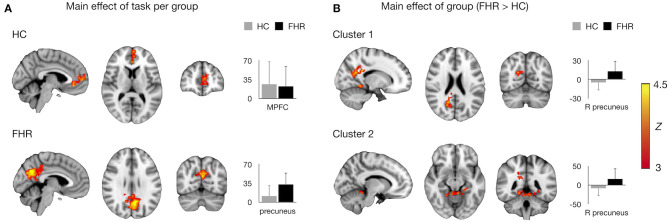
Main effects of task and group on brain activation. **(A)** Mean activation during self-referential processing (self-reference > semantic) per group. MNI coordinates for HC: x = −4, y = −68, x = 28, consistent with MPFC/ACC; for FHR: x = −4, y = 48, z = 10, consistent with precuneus/PCC. **(B)** Significant clusters in the FHR > HC contrast. MNI coordinates for cluster 1: x = 16, y = −64, z = 22, consistent with right precuneus/PCC; cluster 2: x = −10, y = −44, z = 22, consistent with anterior cerebellum/parahippocampal gyrus/lingual gyrus. Significant clusters are overlaid on the MNI template brain. Bar charts depict mean (sd) activation per group.

Assessing main effects of task in each group revealed activation clusters in bilateral MPFC/ACC in HC and precuneus/PCC in FHR (Figure 1A, Table 3).

**Table 3:** Neural activation during self-referential processing.

**Brain regions**	**Side**	**BA**	**MNI**			**Voxels (No.)**	***Z*-max**	***P*-value**
			**x**	**y**	**z**			
**HC**								
MPFC / ACC	L/R	9, 10, 24, 32	−4	36	−8	691	4.18	<0.0001
**FHR**								
Precuneus / PCC	L/R	23, 29, 31	−6	−62	32	1,608	5.34	<0.0001
**FHR** **>** **HC**								
Precuneus / PCC	R	31	16	−40	26	378	4.37	0.004
Anterior cerebellum / Parahippocampal gyrus / Lingual gyrus	L/R	I-V^a^ 27, 35 19	−20	−34	−8	319	4.06	0.009

Activation clusters for self-reference > semantic processing, showing main effect of task for HC and FHR groups, and for the FHR > HC group-contrast. No significant clusters were detected for the HC > FHR contrast.

MNI, Montreal Neurological Institute; BA, Brodmann area.

*^a^For cerebellar regions, we list lobules rather than BA area*.

#### Group-Effects

The FHR > HC contrast yielded two clusters of hyperactivation in FHR. The largest cluster was localized in right precuneus/PCC. A second cluster was found in bilateral anterior cerebellum and inferior temporo-occipital cortex, including parahippocampal and lingual gyrus (Figure 1B, Table 3). No significant clusters were found for the HC > FHR contrast.

#### Validation Analyses

To ensure that effects were not driven by familial ties within subject groups, group-effects were reassessed in a subset including only unrelated individuals (*N* = 26, including 10 FHR and 16 HC), which confirmed increased activation in FHR vs. HC for both the precuneus/PCC (*p* = 0.003) and cerebellar (*p* = 0.019) cluster (see Supplementary Materials and Supplementary Figure 3 for details). In addition, to ascertain that the current results were not driven by the preponderance of ADHD diagnoses in the FHR group, we compared cluster activation levels between FHR with and without an ADHD diagnosis, and between each of these groups and HC, which confirmed that the current results were not accounted for by psychiatric diagnosis (see Supplementary Materials including Supplementary Figure 4).

#### Characterization of Cerebellar Cluster

**Figure 2:**
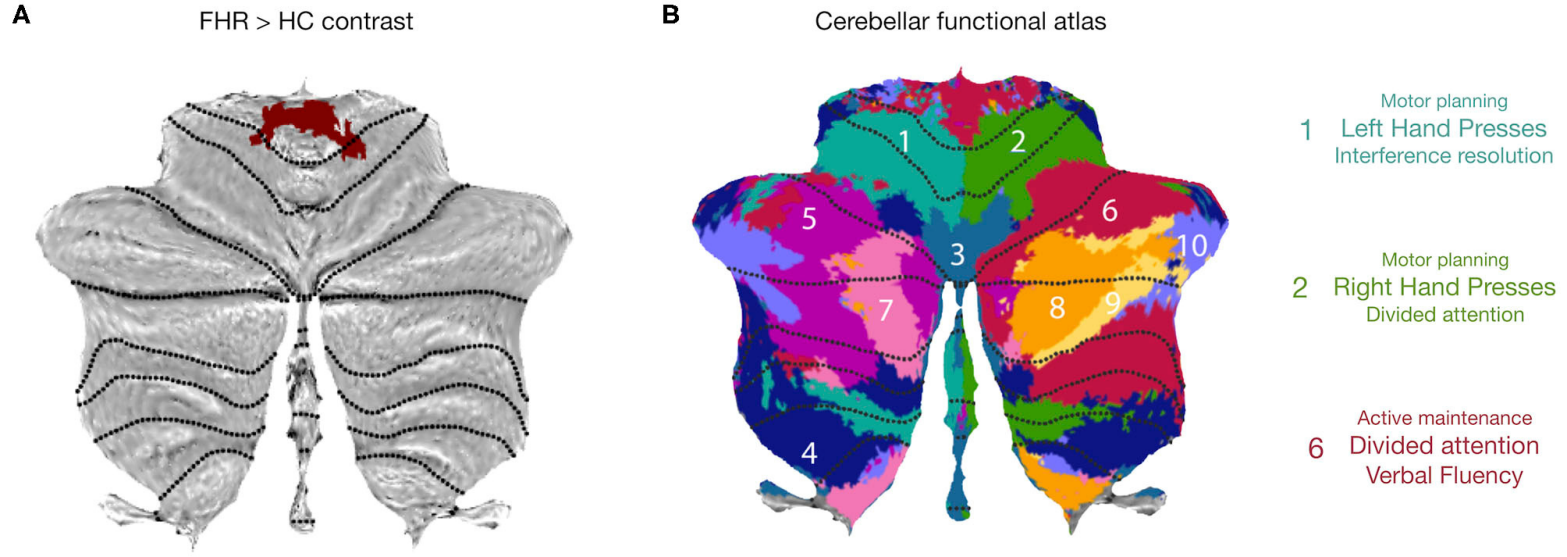
Characterization of cerebellar cluster. **(A)** Cerebellar flatmap showing cerebellar portion of second activation cluster in FHR > HC contrast. **(B)** Cerebellar functional atlas, illustrating that the cerebellar cluster spanned functional regions associated with left and right-hand presses (1,2) and divided attention/verbal fluency (6). **(B)** adapted, *with permission*, from King et al. (2019). Reprint license for **(B)** has been obtained from Nature Neuroscience through RightsLink.

The cerebellar section of the second cluster was projected onto a cerebellar flat map (39) (Figure 2A) and compared to a functional atlas (Figure 2B), which showed that the cerebellar cluster spans areas related to left/right hand presses and active maintenance/verbal fluency.

### Associations With Clinical Measures

**Figure 3:**
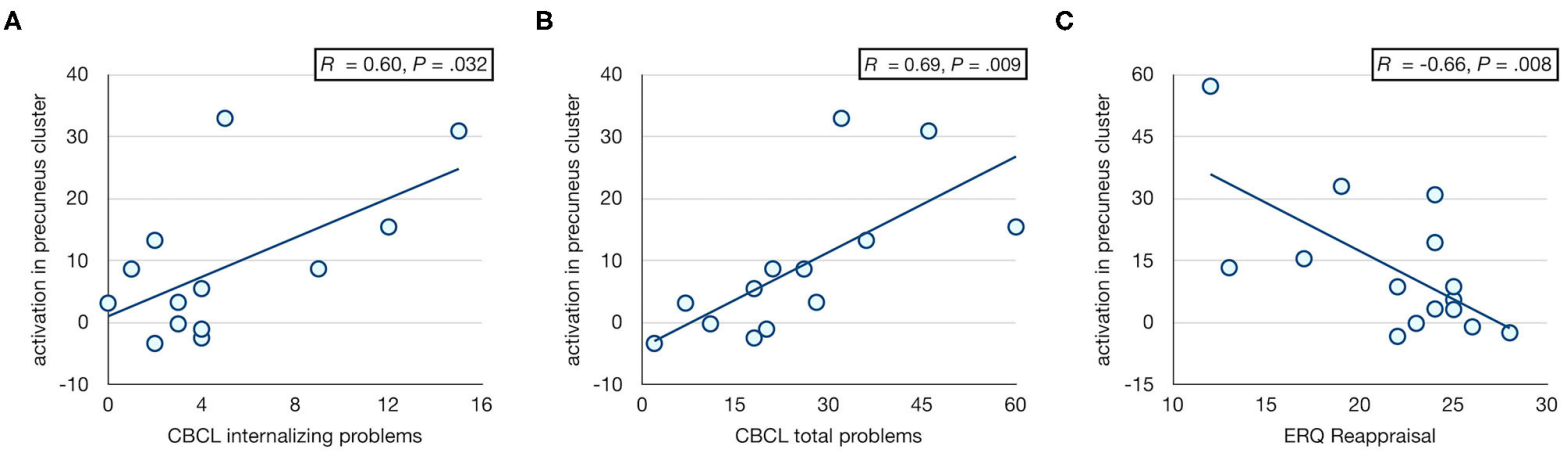
Associations with CBCL scores. Scatter plots showing associations between activation in right precuneus/PCC cluster and CBCL internalizing **(A)** and total **(B)** problem scores, and ERQ cognitive reappraisal **(C)** in FHR. The major CBCL outlier (Supplementary Figure 1) was omitted from the correlation analysis with CBCL scores. Including this participant yielded similar but less significant correlations (i.e., *r* = 0.50, *p* = 0.072 and *r* = 0.56, *p* = 0.039 for internalizing and total problems, respectively).

Activation in the precuneus/PCC cluster was associated with CBCL internalizing and total problems in FHR, such that higher activation was associated with increased internalizing (*r* = 0.60, *p* = 0.032) and total (*r* = 0.69, *p* = 0.009) problems (Figures 3A,B). The cerebellar cluster did not show significant behavioral associations. IQ was not associated with activation in either cluster. Neither cluster showed an association with motion (both *p* > 0.38).

#### Exploratory Analysis

Given the association between precuneus/PCC activation and CBCL total problems, exploratory analyses were performed to assess correlations with CBCL subscale scores. These analyses showed associations with CBCL thought problems (*r* = 0.91, *p* < 0.001), social problems (*r* = 0.70, *p* = 0.005), and withdrawn (*r* = 0.66, *p* = 0.011) subscales (Supplementary Materials, Supplementary Figure 5).

#### Post-hoc Analysis

Following the observation that activation in the right precuneus/PCC cluster was associated with internalizing problems, a *post-hoc* analysis was performed to test for associations with emotion regulation strategies. A significant correlation was observed between precuneus/PCC activation and ERQ reappraisal in FHR, such that more frequent use of cognitive reappraisal as a strategy for emotion regulation was associated with less prominent hyperactivation of precuneus/PCC (*r* = −0.66, *p* = 0.008; Figure 3C).

## Discussion

In this study, we examined functional brain activation related to self-referential processing in children with a familial risk for psychosis. While making judgments on whether or not a list of adjectives described them, in contrast to making a semantic evaluation on whether the words described a good or bad trait, FHR children were found to show hyperactivation of posterior DMN regions including precuneus/PCC as compared to a group of age- and sex-matched HC children. This finding suggests that DMN hyperactivation, as has been observed in adult patients with schizophrenia, dates back at least to middle to late childhood, and thus predates the manifestation of the illness by many years.

The current results are highly consistent with previous literature showing a pattern of anterior DMN (MPFC) hypoactivation and posterior DMN (precuneus/PCC) hyperactivation during self-related processing in (adult) patients with schizophrenia (8–10, 16, 40, 41) and their unaffected relatives (16, 17). Replicating these findings in FHR children confirms and extends these earlier results by showing that DMN-related abnormalities are present in children with a familial risk for psychosis, years before the typical age of onset. Given the young age of our sample, our results indicate that DMN changes also predate adolescence and the maturational brain changes that take place during this developmental epoch. Taken together, our findings thus support hypotheses that abnormal DMN-related self-referential processing is a developmental brain abnormality associated with familial risk for psychosis.

Dissimilar results have also been reported by studies investigating self-referential processing in psychotic illness. For example, van der Meer et al. reported precuneus/PCC *hypo*activation for a self-vs.-baseline contrast in schizophrenia patients (42) and Zhang et al. found similarly located hypoactivations in bipolar disorder patients, with schizophrenia patients showing an intermediate effect (43). Other regions, including the left inferior temporal gyrus, bilateral temporal poles (44), and left lateral frontal cortex (45) have also been implicated. These divergent results may relate to differences in experimental paradigms including baseline conditions (42). In particular, the studies referenced here used either non-valenced statements of general knowledge or a lexical control condition rather than positive vs. negative semantic evaluations as used in e.g., Holt et al. (9) and our current study.

Another important finding of the current study is that hyperactivation of precuneus/PCC in FHR was found to correlate with CBCL internalizing and total problems. This finding is of interest as internalizing problems—e.g., feeling anxious, sad, self-conscious, worrying about the future, and rumination—have been linked to maladaptive (excessive) self-focus and aberrant activity in self-referential brain circuits (46, 47). Clinical staging models of psychotic illness development emphasize that internalizing problems in childhood, followed by depressive symptoms in adolescence, may be the antecedents of later psychopathology ranging from major depression to bipolar disorder and schizophrenia (48, 49). Moreover, exploratory correlational analyses with CBCL subscale scores showed that precuneus/PCC hyperactivation may be associated with thought problems, social problems, and withdrawn behavior. The associations with the thought problems and withdrawn subscales specifically suggest that the observed hyperactivity in posterior DMN may be related to ruminative thinking or basic self-disturbances. In light of the aforementioned studies noting the clinical utility of CBCL thought problems and withdrawn subscales as an adjunctive risk screening measure to aid in early detection of at-risk youth (23, 24), these findings suggest that FHR children who are characterized by more severe internalizing and thought problems and more pronounced hyperactivity of posterior DMN may be at greater risk to develop an affective or non-affective psychotic disorder.

This finding is important as it offers opportunities for early intervention. Mindfulness meditation, for example, has been shown to reduce internalizing symptoms, presumably by promoting a shift from narrative self-focus to present-centered experiential awareness [for review, see (21)]. These changes have been linked to modulations of hyperactive self-referential brain systems (50–52). Consistent with the premise that clinical staging models may improve the logic and timing of interventions in psychiatry (53), using mindfulness-meditation or a similar intervention to address internalizing problems and self-related neural processing deficits in high-risk children may prevent or delay progression to later stages of disorder. Importantly, studies have shown that mindfulness training is both feasible in school-aged children and effective in modulating stress and associated neural systems (54). Moreover, emerging evidence suggests that mindfulness training augmented with real-time fMRI neurofeedback to modulate DMN activity may be effective in reducing symptoms in established psychotic illness (55).

To follow up on the observed association between precuneus/PCC activation and internalizing symptoms, we assessed potential correlations with emotion-regulation strategies. Two strategies that are measured with the ERQ were tested: expressive suppression and cognitive reappraisal. Expressive suppression is the process of inhibiting ongoing emotion-expressive behavior; e.g., holding back tears when feeling sad in public. In contrast, cognitive reappraisal is construing a potentially emotion-eliciting situation in a way that changes its emotional impact. For example, viewing a job interview as an opportunity to find out how well a job would fit their person and vice versa, rather than as a test of one's worth. Out of the two, reappraisal has been found to have a more positive influence on affect, relationships, and well-being, and is viewed as the more adaptive emotion regulation strategy (37, 56). Interestingly, FHR children who endorsed using this strategy more habitually were found to show less precuneus/PCC hyperactivation, consistent with studies showing that overactive self-referential processing may impair top-down emotion regulation (21, 57). Furthermore, the association with cognitive reappraisal may be construed as further evidence that (cognitive) modulation of self-referential brain systems is both possible and potentially beneficial to at-risk individuals.

In addition to the precuneus/PCC cluster, FHR children also showed hyperactivation of a cluster encompassing anterior cerebellum and parahippocampal gyrus. High-risk studies have noted that hyperactivity (58, 59) and volume reductions (60–63) of the parahippocampal cortex predate the onset of psychosis and are most pronounced in at-risk youth who go on to develop full psychosis. Evidence on cerebellar abnormalities predating psychosis is scarce but includes evidence for volume reductions of the anterior cerebellum in high-risk youth (64). Moreover, data from patients with schizophrenia is consistent with hyperactivity and hyperconnectivity of the anterior cerebellum (65, 66). Our current finding of anterior cerebellum / parahippocampal hyperactivity in pre-adolescent FHR children suggests that similar changes observed in patients and high-risk youth predate not just psychosis but also the onset of prodromal symptoms.

This study has a number of limitations to consider when interpreting the current results. The first and main limitation is our sample size, which may have reduced our ability to detect more subtle group-differences. Recruiting children in this age range who have a parent or sibling with a psychotic disorder is difficult, as patients affected by psychosis are less likely to have children (67) and considering the destabilizing effects that psychosis can have on families, especially if it affects (one of) the parents. In addition, as psychotic disorders tend to manifest in late teens or early twenties, most people who are diagnosed with schizophrenia no longer have siblings in the 7–12 years range. As a result, recruiting such a young sample of children with a parent or sibling affected by psychosis is highly challenging and we believe that our data and findings are thus a valuable resource to advance our understanding of trajectories of brain abnormalities in early schizophrenia. Moreover, we note that despite our modest sample size, we found robust effects that survived conservative voxel- and cluster-level thresholds (35). Second, our subject groups were not matched on current DSM diagnosis as 40% of the FHR children had a diagnosis of ADHD with or without co-morbid ODD, while none of the HC children had a DSM-diagnosis. However, it is important to appreciate that childhood developmental disorders are common in FHR individuals and may be linked to (genetic) risk factors for schizophrenia (68). As such, including only FHR without any DSM-diagnoses is likely to produce an overly healthy subset of FHR that may exclude much of the important risk signal. Moreover, direct comparison of FHR with and without current DSM diagnoses did not show group-effects. Third, our processing involved spatial normalization to a standard adult template rather than an age-specific template. The use of age-appropriate templates for normalization is thought to be particularly important in children under 6 years of age (69), but has been shown to improve results in older children as well (70, 71). Important advantages of using age-appropriate templates include reduced individual variation and thus greater statistical power to detect group-differences (71). Although we do not assume that the impact of spatial normalization procedures would affect our groups differentially, we note that the lack of age-specific templates signifies a limitation to our study. Fourth, our results are inherently limited by known confounds of fMRI, including head motion and cardio-respiratory influences. We dealt with these issues to current standards and found no group-differences in motion parameters, but we cannot exclude the possibility that motion and physiological influences impacted our results. Finally, the inclusion of siblings in each subject group may have inflated group-effects. However, reassessing the main findings in a subset of only unrelated individuals confirmed our main results.

In summary, the results of this study in a unique sample of FHR children between 7 and 12 years of age suggest that hyperactivity of posterior DMN during self-referential processing develops many years before the typical manifestation of psychosis in association with familial (possibly reflecting genetic) risk factors. Moreover, our study links aberrant self-referential processing to increased levels of internalizing problems and less frequent use of cognitive reappraisal for emotion regulation. These findings have implications for our understanding of the developmental timeline of DMN abnormalities in schizophrenia spectrum disorders and may inform strategies for early intervention: aiming to reduce internalizing problems by modulating DMN-related self-referential processing in the early premorbid stage.

## Data Availability Statement

The data analyzed in this study is subject to the following licenses/restrictions: the raw data from this study is not publicly available because participants did not agree for their data to be shared publicly. De-identified processed data that support the findings of this study are available from the corresponding author, upon reasonable request. Requests to access these datasets should be directed to gcollin@mit.edu.

## Ethics Statement

This study involved human participants and was reviewed and approved by the Institutional Review Board (IRB) of Beth Israel Deaconess Medical Center (BIDMC), Boston, MA, USA. Written informed consent to participate in this study was provided by the participants' parents/legal guardians and participants provided assent to participate in the study.

## Author Contributions

LS, MK, JG, HT, and SW-G contributed to conception and design of the study, and the acquisition of funding. EM, RM-G, and HT contributed to data acquisition. LS, RM-G, and MK led clinical consensus meetings. GC and CB performed the statistical analysis. GC wrote the first draft of the manuscript. CB wrote sections of the manuscript. SA contributed to the cerebellar analyses. GC, CB, SA, MS, and SW-G contributed to data interpretation. All authors contributed to manuscript revision, read, and approved the submitted version.

## Conflict of Interest

The authors declare that the research was conducted in the absence of any commercial or financial relationships that could be construed as a potential conflict of interest.
