# Supplementary material for: Hyperactivation of Posterior Default Mode Network During Self-Referential Processing in Children at Familial High-Risk for Psychosis
Source: Front Psychiatry. 2021 Feb 9;12:613142. doi: 10.3389/fpsyt.2021.613142 (PMC7900488; doi:10.3389/fpsyt.2021.613142)
Supplement: Supplementary file 1 [file Data_Sheet_1.docx]

**Supplementary Material**

**CBCL outlier**

There was one major outlier in CBCL internalizing, externalizing, and total problems (all > 3 standard deviations from the mean) (**Supplementary Figure 1**). This participant was omitted from correlational analyses with fMRI results.

**SUPPLEMENTARY FIGURE 1 |** CBCL outlier. Boxplots depicting CBCL scores for internalizing, externalizing and total problems across the whole sample, showing one major outlier (#27 in the plot).

**Behavioral correlations of task performance and self-appraisal**

In FHR participants, IQ was significantly associated with semantic performance (*r* = 0.63, *p* = 0.013) and negative self-appraisal (*r* = -0.59, *p* = 0.018) (**Supplementary Figure 2**), suggesting that high-risk children with lower IQ performed worse during the semantic condition and associated more negatively-laden words with ‘me’.

**SUPPLEMENTARY FIGURE 2 |** Associations of behavioral results with IQ. Scatter plots illustrating the association between WISC IQ and semantic performance **(left panel)** and negative self-appraisal **(right panel)**, which were significantly associated in FHR. Semantic performance was computed as the percentage of correct responses on the semantic condition (i.e. determining if a presented word signified a good or bad trait). Negative self-appraisal was measured as the percentage of semantically bad adjectives associated with ‘me’.

**Impact of family ties**

The current study included a total of 37 participants, of whom 34 (15 FHR, 19 HC) were included in final analysis. This final sample included three sibling pairs and one set of three siblings in the FHR group and 3 sibling pairs in the HC group. To ascertain that the main results were not driven by family ties within groups, results were reassessed in a subsample of unrelated individuals (10 FHR, 16 HC; demographic and clinical information in **Supplementary Table 1**). Group-comparison in this subsample confirmed increased activation in FHR versus HC for both the precuneus/PCC (*p* = 0.003) and cerebellar (*p* = 0.019) cluster (**Supplementary Figure 3**).

**Supplementary Table 1 |** Demographic and clinical information of unrelated subsample

|  | | **FHR**  **(*N* = 10)** | **HC**  **(*N* = 16)** | **Statistics** |
| --- | --- | --- | --- | --- |
| Age in years, mean (sd) [range] | | 9.4 (2.0)  [7.0 – 12.0] | 9.2 (1.6)  [7.2 – 11.8] | *F_(1,33)_* = 0.1, *p* = 0.79 |
| Sex, M/F | | 4 / 6 | 7 / 9 | *χ^2^* = 0.04, *p* = 0.85 |
| WISC IQ, mean (sd) [range] | | 101.7 (16.6)  [73 – 132] | 112.9 (16.4)  [84 – 153] | *F_(1,33)_* = 2.8, *p* = 0.11 |
| DSM-diagnosis | |  |  | *χ^2^* = 7.57, *p* = 0.056 |
|  | ADHD | 2 | 0 |  |
|  | ADHD/ODD | 2 | 0 |  |
|  | No diagnosis | 4 | 11 |  |
|  | Data missing | 2 | 5 |  |

*Statistical comparisons: ANOVA for continuous and chi-squared tests for categorical variables. ADHD, attention deficit hyperactivity disorder; ODD, oppositional defiant disorder.*

**SUPPLEMENTARY FIGURE 3 |** Significant group-effects in subset of unrelated participants. Bar charts showing mean activation in precuneus/PCC **(left panel)** and cerebellar **(right panel)** cluster during self-referential processing in unrelated sample of HC (*N* = 16) and FHR (*N* = 10) participants, illustrating significantly increased activation in FHR for both clusters.

**Impact of ADHD diagnosis**

DSM-diagnoses were significantly more common in FHR than HC participants (i.e., 6 FHR diagnosed with ADHD with/without comorbid ODD; versus no DSM diagnoses in the HC group, *p* = .025). To ascertain that group-effects were not better accounted for by ADHD diagnosis than FHR status, we compared cluster activation levels among three groups: HC, FHR with ADHD (FHR_ADHD_), and FHR without a DSM diagnosis (FHR_no-diagnosis_).

Inspection of the data (Figure S4) gave no indication of increased precuneus/PCC or cerebellar activation in FHR_ADHD_ as compared to FHR_no-diagnosis_, which was confirmed with independent samples t-tests (*p* = .89 for precuneus/PCC and *p* = .67 for cerebellar cluster). Moreover, both FHR groups showed increased activation relative to HC for both the precuneus (*p* = .018 for FHR_no-diagnosis_ and *p* = .004 for FHR_ADHD_) and cerebellar (*p* = .032 for FHR_no-diagnosis_ and *p* = .053 for FHR_ADHD_) cluster.

**Figure S4. Group-effects in HC, FHR with ADHD, and FHR without ADHD**

Bar charts showing mean precuneus/PCC and cerebellar activation in HC, FHR without a DSM diagnosis, and FHR diagnosed with ADHD (with/without ODD), confirming main results.**Exploratory analysis with CBCL subscale scores**

Precuneus/PCC activation was found to be associated with CBCL total problems. To further examine this association, exploratory correlational analyses were performed to assess precuneus/PCC activation for associations with CBCL subscale scores (Figure S5).

**Figure S5. Correlations between precuneus/PCC activation and CBCL subscale scores**

Scatter plots showing associations between precuneus/PCC activation and eight CBCL subscale scores. Statistics for correlations at uncorrected *p* < 0.05 are in bold print (i.e., thought problems, social problems, and withdrawn subscales).
